# Commentary: Mechanisms of mitral valve development and disease

**DOI:** 10.3389/fcvm.2026.1885698

**Published:** 2026-06-23

**Authors:** Giulio Calcagni, Flaminia Pugnaloni, Paolo Versacci, Bruno Marino

**Affiliations:** 1 Clinical Cardiology Unit, Division of Cardiac Surgery, “Bambino Gesù” Children's Hospital IRCCS, Rome, Italy; 2 Neonatal Intensive Care Unit, “Bambino Gesù” Children's Hospital IRCCS, Rome, Italy; 3 Department of Maternal Infantile and Urological Sciences, Sapienza University of Rome, Rome, Italy

**Keywords:** congenital heart defect, genetic syndromes, hypertrophic cardiomyopathy, mitral valve, RASopathies, down syndrome

We read with great interest the review by Wang and Zhou on the mechanisms of mitral valve (MV) development and disease (1). Their article provides a valuable and elegant synthesis of developmental biology and pathology, linking embryologic mechanisms to three major mitral phenotypes: rheumatic mitral stenosis, congenital mitral stenosis, and myxomatous mitral valve prolapse. Notably, across each of the three conditions examined, the authors develop their analysis from the embryologic basis of MV formation to the mechanisms responsible for disease onset and progression, including endothelial injury as a putative etiologic factor, the contribution of inflammatory pathways, and the genetic predisposition underlying myxomatous degeneration (1).

In addition to the forms of MV disease addressed in the review, we believe that MV abnormalities in patients with genetic syndromes represent an equally important group that deserves explicit consideration. These conditions offer a clinically and biologically informative details into the relationship between altered developmental signalling, atrioventricular cushion morphogenesis, extracellular matrix remodelling, and final valve phenotype.

From a developmental standpoint, these observations may be interpreted in light of the role of the dorsal mesenchymal protrusion, a second heart field–derived structure crucial to atrioventricular canal formation, whose development is influenced by Sonic Hedgehog signalling and by laterality-related pathways involving *PITX2* (2). This perspective may be especially relevant in Down syndrome, in which cardiac disease is strongly associated with atrioventricular septal defects (AVCD) and abnormal atrioventricular junction development (2, 3). In this setting, Sonic Hedgehog signalling is of particular interest, since Hedgehog-dependent mechanisms have been proposed as a unifying pathway in AVCD associated with genetic syndromes, including Down syndrome (2). Accordingly, abnormalities involving the left atrioventricular valve/mitral valve apparatus may be viewed not merely as secondary components of a complex defect, but as part of a broader developmental spectrum linking altered atrioventricular patterning to clinically relevant mitral phenotypes.

A similar developmental perspective emerges in RASopathies, a group of disorders caused by dysregulation of the RAS/MAPK signalling pathway. Although their cardiovascular phenotype is classically dominated by pulmonary valve stenosis, hypertrophic cardiomyopathy (HCM), and septal defects, MV involvement is increasingly recognized. Reported abnormalities include mitral regurgitation, leaflet dysplasia, prolapse, and, in some cases, stenotic lesions (4, 5).

Updated data from the CARNET experience further support the relevance of mitral involvement, showing that atypical valvular disease in these patients frequently affects the MV and that regurgitant lesions are more common than stenotic ones (4). In addition, within the broader spectrum of HCM, the MV is often structurally abnormal and appears to participate directly in the disease process rather than acting as a merely secondary hemodynamic bystander (5).

In a landmark imaging study, Maron et al. showed that mitral leaflet elongation in HCM is independent of other disease variables and may represent a primary phenotypic expression of the disease (6).

Conversely, other syndromic conditions further emphasize the MV valve as a clinically relevant expression of altered developmental programs, reflecting the heterogeneous genetic and morphogenetic mechanisms that shape atrioventricular and valvular phenotypes.

In molecularly confirmed KBG syndrome, congenital heart defects include MV abnormalities, further reinforcing the view that syndromic disorders may offer a broader developmental perspective on MV disease (7, 8).

Finally, the spectrum may be further extended to other syndromic conditions, such as in the spectrum of collagenopathies (Loeys–Dietz or Marfan syndrome), in which MV involvement as a marker of heritable connective tissue disorders, likewise highlights the heterogeneous developmental and structural pathways leading to mitral pathology (9).

In conclusion, we believe that MV disease in the setting of genetic syndromes may further expand the framework proposed by the authors, highlighting how defined genetic and developmental contexts contribute to the heterogeneity of valve pathology explicitly. These conditions may further strengthen developmental models of disease and broaden their relevance to clinically complex and genetically defined patient populations.
